# Stepping up ELISpot: Multi-Level Analysis in FluoroSpot Assays

**DOI:** 10.3390/cells3041102

**Published:** 2014-11-27

**Authors:** Sylvia Janetzki, Markus Rueger, Tomas Dillenbeck

**Affiliations:** 1 ZellNet Consulting, Inc., 555 North Avenue, Suite 25-S, Fort Lee, NJ 07024, USA; 2 Autoimmun Diagnostika GmbH, Ebinger Strasse 4, D-72479 Strassberg, Germany; E-Mail: rueger@aid-diagnostika.com; 3 Mabtech AB, Box 1233, SE-131 28 Nacka Strand, Sweden; E-Mail: Tomas.Dillenbeck@mabtech.com

**Keywords:** FluoroSpot, ELISpot, immunomonitoring, image analysis, fluorochromes, fluorophores

## Abstract

ELISpot is one of the most commonly used immune monitoring assays, which allows the functional assessment of the immune system at the single cell level. With its outstanding sensitivity and ease of performance, the assay has recently advanced from the mere single function cell analysis to multifunctional analysis by implementing detection reagents that are labeled with fluorophores (FluoroSpot), allowing the detection of secretion patterns of two or more analytes in a single well. However, the automated evaluation of such assays presents various challenges for image analysis. Here we dissect the technical and methodological requirements for a reliable analysis of FluoroSpot assays, introduce important quality control measures and provide advice for proper interpretation of results obtained by automated imaging systems.

## 1. Introduction

After its first description in 1983 [1], the solid-phase enzyme-linked immunospot (ELISpot) assay became one of the most commonly used immune monitoring assays in basic and translational research in many fields of immunology [2]. In addition to numerous research applications, it is nowadays also applied in clinical trials to search for biomarkers indicative of the success of immunotherapeutic interventions [3], or even as a diagnostic tool [4]. The importance of the ELISpot assay is further underlined by the wealth of conducted and ongoing projects that aim at the quality of assay performance and its reproducibility across laboratories [5,6,7,8,9,10,11]. Established ELISpot harmonization guidelines have reduced the variability in reported results [12], and proficiency panel testing is now available for any laboratory seeking an external validation of their assay conduct [13].

**Figure 1:**
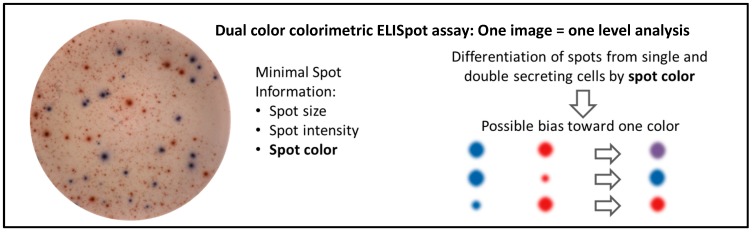
Color bias in colorimetric dual color ELISpot assay. Evaluation of enzymatic dual color ELISpot assays is solely based on spot color, with single cytokine spots being red or blue, and dual cytokine spots theoretically purple. Dual spots with high intensity in only one color risk being misinterpreted as single spots as the stronger spot will obscures the weaker one. This will result in an underestimate of the number of dual secreting cells as well as an underestimate of the total number of spots for the weaker color.

The widespread use of ELISpot over the past decades can mainly be attributed to its outstanding sensitivity, ease of implementation, and robustness. Further, the technique itself varies only little, if at all, for the analysis of a great variety of cytokines, antibodies, and other secreted proteins, e.g., chemokines or apolipoproteins [14,15]. Hence it was only a question of time until the polyfunctional analysis capabilities of ELISpot were addressed by looking at the simultaneous secretion of two analytes in one well. This was originally attempted with the establishment of dual color enzymatic ELISpot assays [16]. Plates were coated and developed with two antibody pairs with affinities for different cytokines, e.g., IFNɣ and IL-2 [17]. Spots were developed using two different combinations of enzymes and substrates (e.g., alkaline phosphatase with a blue colored spot-forming chromogenic substrate for the first analyte, and horseradish peroxidase with a red spot-forming substrate for the second). Such assays opened the possibility to determine the number of cells secreting either one of the two analytes or both analytes simultaneously by using spot color to differentiate between the three cell populations. Double secreting cells would produce purple colored spots while single secreting cells would give blue or red spots. However, it became evident early on that such spots are difficult to interpret [2,16] as their color may range across all shades of purple, from completely red to completely blue. Specifically the problem lies in a bias towards single secretion for dual colored spots with a considerable difference in size and/or intensity, where a weaker spot in one color is easily obscured by a stronger spot in a second color (Figure 1). This can lead to an underestimation of the number of double spots, but also an underestimation of the total number of spots in each color as faint double spots may be missed completely.

The introduction of the FluoroSpot assay [18,19], with visualization of spots by fluorophores instead of enzyme and substrate combinations, led to an improvement in the detection of double stained spots [19]. But still, at that time assay analysis by automated spot-counting systems was based on spot color. Only with the introduction of a new, two level image analysis technology, an unambiguous differentiation between single and double (or even triple) stained spots became possible. We provide here a detailed description of the improved analysis technology for accurate FluoroSpot evaluation, technical requirements related to fluorophores and the automated imaging system, important controls for deeming an imaging system suitable for FluoroSpot evaluation, and an assessment of results obtained with automated evaluation of FluoroSpot assays.

## 2. Experimental Section

This section is written in compliance with the MIATA guidelines for transparent reporting [20].

*The Sample:* Peripheral blood mononuclear cells (PBMC) were prepared by Ficoll density centrifugation of buffy coats obtained from heparinized blood from two anonymous healthy blood donors at Karolinska Hospital, Solna, Sweden. The time frame between blood draw and PBMC isolation averaged 20 h. PBMC were frozen at −80°C in 20% fetal calf serum (FCS)/10% dimethyl sulfoxide (DMSO) using a Mr. Frosty device, and transferred and stored in liquid nitrogen until further use (within < 1 year). PBMC were thawed and washed twice before left to rest for two hours at 37°C and 5% CO_2_. Cell concentration and viability were determined by the Guava ViaCount assay (Guava Technologies, Hayward, CA, USA). Viability was >90%.

*The Assay*: RPMI 1640, HEPES, penicillin/streptomycin, and low endotoxin FCS were purchased from Invitrogen Life Technologies (Carlsbad, CA, USA). The serum lot was pretested to check for adverse effects. Pre-coated human IFNɣ/IL-2 FluoroSpot plates (monoclonal antibodies (mAbs) 1-D1K and MT2A91/2C95), pre-coated human IFNɣ /IL-22/IL-17A FluoroSpot plates (mAbs 1-D1K, MT12A3, and MT44.6), detection reagents: 7-B6-1-FS-FITC, 7-B6-1-biotin, 7-B6-1-BAM, MT8G10-biotin, MT7B27-biotin, MT504-BAM, anti-FITC-490, streptavidin-550 (SA-550), anti-BAM-640, and fluorescence enhancer, as well as stimuli: CEF peptide pool, anti-CD3 mAb, and anti-CD28 mAb, were all obtained from Mabtech, Nacka Strand, Sweden (Mabtech kit for IFNɣ/IL-2: cat# FSP-0102, and for IFNɣ/IL-22/IL-17A: cat# FSP-011803). The FluoroSpot plates, which contain a low auto-fluorescent PVDF membrane, were originally obtained from Millipore (cat# S5EJ104I07). *Candida albicans* extract was obtained from Greer, Lenoir, NC, USA. Pre-coated human IFNɣ/IL-2 FluoroSpot plates were washed five times with 200 µL/well sterile phosphate-buffered saline (PBS), and blocked for 1 h with 200 µL/well cell culture medium (RPMI 1640 supplemented with 10% heat-inactivated FCS, 1 mM glutamine, 100 units/mL penicillin, 100 µg/mL streptomycin and 0.5 mM HEPES). The blocking medium was removed and 100 µL/well of new medium with 0.1 µg/mL anti-CD28 mAb (to counter-act the absorption effect of IL-2 that leads to decreased costimulation and potentially lower IFNɣ spot counts), with or without stimuli (2 µg/mL CEF) added to each well. Rested PBMC were added at 250,000 cells in 100 µL to each well, with each sample and condition analyzed in triplicates. The plates were then incubated for 20 h at 37 °C and 5% CO_2_. The following day, the cells were removed by washing five times with PBS (200 µL/well) in an automated ELISA washer (Bio-Tek Instruments Inc., Winooski, VT, USA). For single stained wells detection antibodies conjugated with FITC, biotin, or BAM peptide were individually diluted in PBS with 0.1% BSA (PBS/BSA) to 1 µg/mL, and 100 µL were added to each well for two hours of incubation at room temperature (RT). Plates were then washed five times as described above before the addition of one of the secondary reagents: anti-FITC-490, SA-550, or anti-BAM-640 (each diluted 1:200 in PBS/BSA), followed by an one hour incubation at RT. Plates were again washed as described above, and 50 µL/well of fluorescence enhancer added for a 15 min incubation. Plates were emptied thoroughly and the underdrain removed before leaving the plates to dry protected from light.

For IFNɣ /IL-2 dual FluoroSpot, anti-IFNɣ (7-B6-1-FS-FITC, diluted 1:200) and anti-IL-2 (MT8G10-biotin, diluted to 1 µg/ML) detection mAbs were together added to each well in 100 µL PBS/BSA for a two hour incubation at RT. After washing, anti-FITC-490 and SA-550 (both diluted 1:200) were added to all wells and incubated for one hour at RT.

For IFNɣ/IL-22/IL-17A triple FluoroSpot, 300,000 PBMC were seeded per well and incubated over two nights with or without *Candida albicans* extract (20 µg/mL), considering the slower secretion kinetics especially for IL-17A with the given stimulating agent. On day three the cells were washed away as described above, and anti-IFNɣ (7-B6-1-FS-FITC, diluted 1:200), anti-IL-22 (MT7B27-biotin, diluted to 0.5 µg/mL), and anti-IL-17A (MT504-BAM, diluted 1:200) detection mAbs were mixed and 100 µL added to each well for two hours incubation at RT. The plates were washed and the secondary reagents: anti-FITC-490, SA-550, and anti-BAM-640, were all diluted 1:200 and added to all wells for one hour incubation at RT.

*Data Acquisition*: Analysis and counting of spots were done with a FluoroSpot reader system (iSpot Spectrum, AID, Strassberg, Germany) with software version 7.0, build 14790, where fluorescent spots were counted utilizing separate filters for FITC, Cy3, and Cy5. Camera settings (Exposure and Gain) were adapted for each filter to obtain high quality spot images preventing over- or underexposure. Fluorophore-specific spot parameters were defined using spot size, spot intensity and spot gradient (fading of staining intensity from center to periphery of spot); and a spot separation algorithm was applied for optimal spot detection. Parameters were fine-tuned comparing negative control wells (PBMC alone) and wells containing CEF-stimulated PBMC. Additional images were taken by a Zeiss ELISpot reader (Carl Zeiss, Inc., Thornwood, NY, USA) equipped with a double band fluorescence filter for FITC and Rhodamine for fluorescent signal detection in one image by color discrimination, using KS ELISpot 4.9 software.

*Data and Response determination:* Raw data can be made available upon request. No statistical analysis or response determination was performed for this study.

*Lab Operations*: The experiments were performed in accordance with established Standard Operating Procedures (SOPs) and with validated reagents in an investigative laboratory that operates in accordance with ISO 9001/ISO 13485. The laboratory participates in ELISpot proficiency panels conducted by the Cancer Immunotherapy Consortium (CIC), the Association for Cancer Immunotherapy (CIMT) and Immudex.

## 3. Results and Discussion

### 3.1. The Multi-Dimensional FluoroSpot Analysis

The introduction of a new approach for FluoroSpot analysis was driven by the need to overcome the limitations of spot analysis by color (Table 1).

**Table 1:** Summary of the pros and cons for automated evaluation of different ELISpot assay formats.

	ELISpot	Dual ELISpot	FluoroSpot
**Assay Qualities**			
Signal stability	+	+	+
Assay sensitivity	+	+	+
High-throughput	+	(+)	+
Amount of data generated per sample	+	+ +	+ + +
Robustness	+	−	+
**Level of Analysis**			
Simultaneous analytes	−	+	+ +
Subpopulation analysis	−	(+)	+
**Reader Compatibility**			
Standard ELISpot reader	+	+	−
Standard FluoroSpot reader	+	+	+
(Filter sets, strong light source, and multi-level image analysis)			

− not possible or applicable; (+) limited; + good or applicable; ++ very good; +++ outstanding.

**Figure 2:**
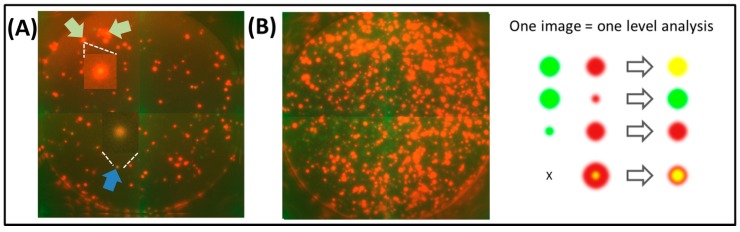
Challenges of broadband filter analysis of FluoroSpot plates. PBMC were stimulated with the CEF peptide pool (**A**) or anti-CD3 (**B**) and tested simultaneously for IFNɣ and IL-2 secretion using FITC (for IFNɣ) and Cy3 (for IL-2) fluorophores. Images were taken with a Zeiss reader utilizing a dual band filter for FITC and Rhodamine. FITC signals are weak and small, while Cy3 signals are strong and larger, overlaying smaller FITC spots (**B**), leading to false low counts for green spots. In panel (**A**), the blue arrow points to a yellow spot that is likely to be caused by dual secretion of cytokine by one cell (see insert for close up). Similar yellow signals can also be found in the center of larger, high intensity red spots (green arrows), leading to false high dual spot counts (see insert for close-up). The challenges of such one level FluoroSpot analysis are illustrated on the right.

Analysis by color can lead to either falsely defined single color spots (the fainter spot is simply not distinguishable due to overlay by the stronger spot), or to falsely defined dual colored spots when the larger spot partially overlays a smaller spot that is located in its periphery, but which is produced by a different cell (Figure 2). Further, spots of very high intensity (e.g., for Cy3, typically dark orange to red with most broadband filters) may appear with a yellow center, wrongly implying a dual colored spot (Figure 2). Lastly, since camera settings cannot be set individually for two fluorophores when using a dual band filter, spots of one color (e.g., green spots for FITC) may appear as weak signals while spots of another color (e.g., orange-red spots for Cy3) may be overexposed (Figure 2).

**Figure 3:**
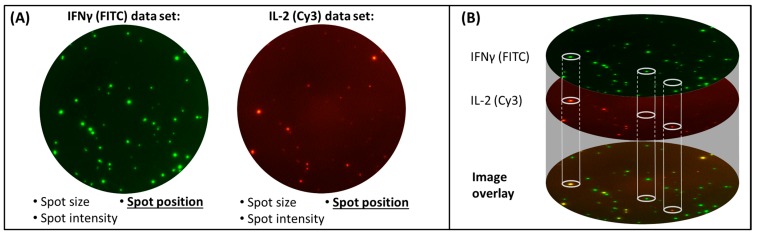
Two level FluoroSpot analysis. PBMC were stimulated with CEF peptide pool and tested simultaneously for IFNɣ and IL-2 secretion using FITC (for IFNɣ) and Cy3 (for IL-2) fluorophores. Images were taken with an AID Imaging Analyzer utilizing narrow band filters for each fluorophore. Panel (**A**) demonstrates the two separate images taken of the same well (Level one analysis). Spots are analyzed for e.g., spot size, spot intensity and location. Images are then superimposed and a location algorithm is applied that allows the identification of spots resulting from single and dual cytokine-producing cells based on the exact location parameters of each spot center (Level two analysis, panel (**B**)).

The key innovation introduced for FluoroSpot plate evaluation is a two level analysis of every well (Figure 3). On the first level, separate images of each analyte/fluorophore are acquired and analyzed. In case of a dual color FluoroSpot, two separate images are taken; in case of triple color FluoroSpot, three separate images are taken *etc.* Importantly, camera settings (e.g., Exposure, Gain) can be adjusted for every analyte/fluorophore to compensate for different fluorescent intensities.

**Figure 4:**
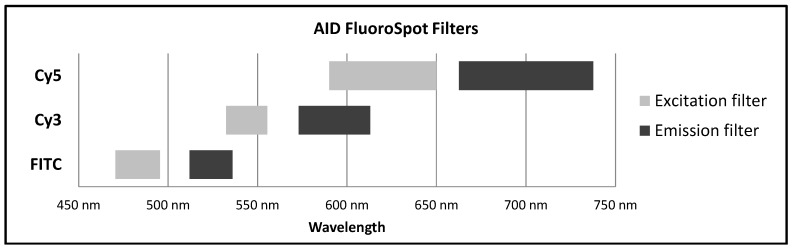
**Excitation and Emission ranges for selected narrow band filters.** A selection of narrow band filters as used in the AID Imaging Analyzer for the evaluation of FluoroSpot assays is depicted. Of note, these filters provide filtration on two levels: 1. Filtering of the incoming excitation light to prevent excitation of fluorophores with partial spectral overlap, and 2. Filtering of emitted signals to obtain defined spot images.

Two prerequisites are essential for successful FluoroSpot evaluation:

1. Narrow band filters with specific excitation and emission wavelength range for each fluorophore to avoid bleed-over between different fluorophores (Figure 4);
2. Software features for the identification of the exact position of each spot in a two dimensional coordinate system.

At level one, the analysis of each image obtained per well is done separately with analyte/fluorophore specific parameters as defined by the user following lab-specific or SOP-defined steps (also see Materials and Methods section, Data Acquisition).

**Figure 5:**
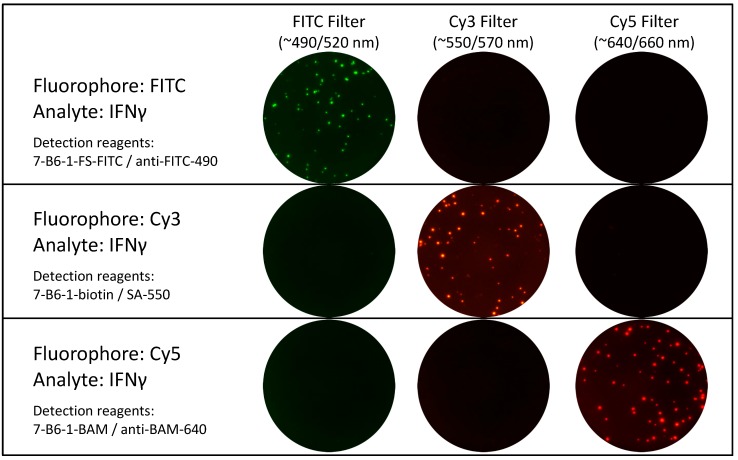
Testing of FluoroSpot imaging systems for signal bleed-over. PBMC were stimulated with CEF and tested for IFNɣ secretion using different fluorophore-labeled detection systems. The level one images for each detection system and filter are shown (Top row: FITC spots with all filters; Middle row: Cy3 spots with all filters; Lower row: Cy5 spots with all filters). Only with the appropriate filter spots are detected. There are no signals apparent for the same well when other filters are used.

**Figure 6:**
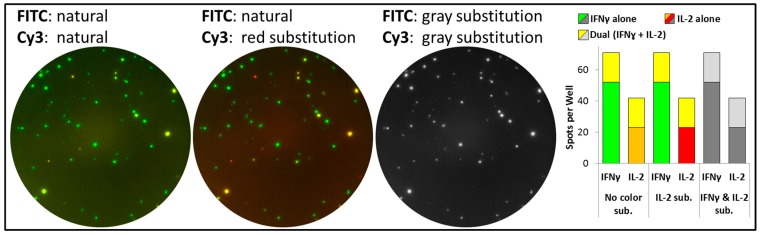
Influence of spot color on spot counts. Overlay images of one well of PBMC stimulated with CEF peptide pool stained for IFNɣ (FITC) and IL-2 secretion (Cy3) are shown with natural appearing colors (left image), color substitution for Cy3 spots (center image) and substitution of both FITC and Cy3 with gray values (right image). Spot counts for each cytokine in each of the three color schemes are presented in the graph, indicating that independent of color adjustments to spots, overall evaluation results are the same for each cytokine and for dual secretion.

**Figure 7:**
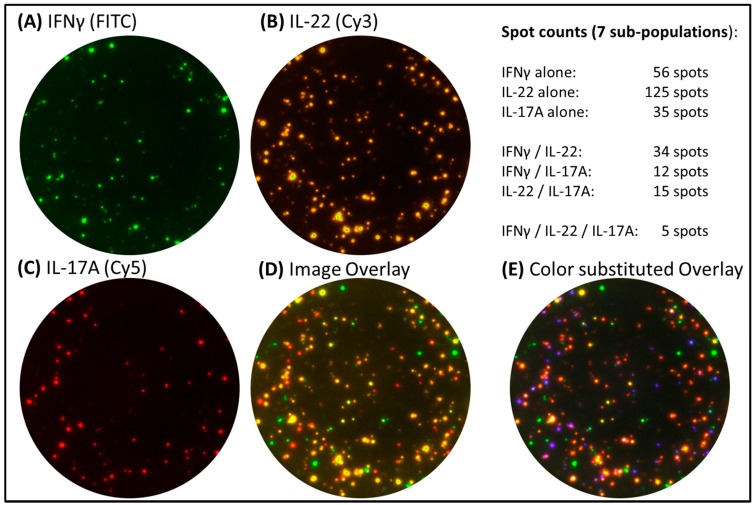
Triple cytokine FluoroSpot assay. PBMC were stimulated with *Candida albicans* extract and tested for IFNɣ (FITC), IL-22 (Cy3) and IL-17A (Cy5) secretion. Level one images for separate fluorophores are depicted in panel (**A**)–(**C**). The level two image overlay and color substituted overlay for visualization purposes are shown in panel (**D**) and (**E**), respectively. The overall spot counts for all seven subpopulations are presented.

During the second level of analysis, the positions of counted spots in the separate images are compared by a location algorithm. If spots in different images (for different fluorophores) of the same well have identical positions as defined by the location of the spot center they are detected as multi-stained spots, otherwise they are counted as single stained spots of the respective analyte/fluorophore (Figure 3). For this approach, it is important that the automated reader system i. locks in the plate at a specifically designated well position, and ii. allows an automated switch of filters as defined by the fluorophores used in the assay. The imaging software applied should further allow the definition of maximum acceptable space between centers of spots detected for different fluorophores for the identification of multi-colored spots. In other words, the different kinetics of cytokine secretion (related to onset, speed, duration, strength) as well as cell movement during incubation may cause a minimal shift of spot centers for a given analyte (typically in the µm range) that needs to be taken into consideration for cells secreting more than one analyte upon stimulation. Technical causes have to also be considered, which can, for example, be related to the image alignment. Rebhahn *et al.* have addressed this topic in detail previously [21]. The authors show that, likely due to the reasons given above, a spot is not perfectly round. Different circularity values for overlaying spots, as determined by measurements of the radial variability (distance of the pixels with highest intensity [namely the spot center mass] to all pixels distributed in its periphery), consequently lead to slight shifts of spot centers. They further address the random overlay of spots by determining a coincidence limit using matched and unmatched image overlays. In our experiments, it was empirically determined that for medium sized spots as apparent in Figure 3, Figure 5, Figure 6 and Figure 7, a pixel shift with the AID Spectrum reader of app. five pixels (roughly 15 µm) works well for capturing the true dual secretors while keeping the rate of random overlay at a minimum.

### 3.2. Fluorophores and Testing of Imaging Systems for Signal Bleed-Over

Currently there are only a limited number of fluorophores used in FluoroSpot, and their selection is mainly driven by the goal of obtaining efficient signal strength and stability while being able to prevent bleed-over signals, also see [22]. The most commonly used fluorophores are FITC, Cy3 and Cy5. A significant overlap of excitation and emission spectra exists for these three fluorophores. However, the use of narrow band filters for the excitation as well as emission can efficiently prevent bleed-over (Figure 4).

It is advisable to test if the automated imaging system chosen for FluoroSpot analysis does indeed prevent signal bleed-over and with that offers accurate plate analysis capabilities. This is efficiently achieved, for example, by using only one fluorophore per well for the detection of cytokine secretion, but evaluating each well with all filters. No signal should be detected when using any filter other than the one designated for the used fluorophore (Figure 5).

### 3.3. The True Meaning of Color in FluoroSpot Analysis

It is important to realize that - different to colorimetric ELISpot assays and FluoroSpot analysis by color (see Figure 1 and Figure 2) - spot color in the captured image is of no importance to FluoroSpot analysis. Fluorescent signals are separated by filters (as discussed above), and image color will only affect the graphical visualization of data. The analysis of FluoroSpot assays as described for this two level technology uses gray values of spots for their identification, and is based on spot parameters as spot size, intensity and position in the first level of analysis. In imaging processing it is a common approach to calculate the intensities of objects across a picture by converting it to a grayscale. The translation of an 8-bit grayscale image into a binary image gives 256 possibilities (from 0 to 255, with 0 being black and 255 being white). Image color in FluoroSpot assays has a pure visualization aspect and colors may be substituted by the software user based on personal preferences to provide a clearer and more intuitive presentation of different spot types or cell populations (see Figure 6 and Figure 7). Caution is advised for existing reports about more than three or four color Fluorospot capabilities, which may simply refer to the different subpopulations detectable, which are marked with different colors by the operator using appropriate software features (see also Figure 7E).

### 3.4. Discussion

The introduction of detection systems using fluorophores for the ELISpot technique has opened the door to the successful analysis of multiple secreted analytes within one well. For data acquisition, existing imaging systems have to be equipped with a strong light source and special filters. It became evident rather fast that evaluation of FluoroSpot plates based on color differentiation of spots provided insurmountable challenges. The solution was found in a two level analysis approach during which a separate image is taken for each fluorophore, the image evaluated for fluorophore-specific spots and the spot location recorded. The separate images for the same well are then checked for spots with the same coordinates to define single and dual (or more) secretors. This approach has already been successfully applied in various fields, like Tuberculosis research [23], HIV research [24], research related to other infectious diseases [25], as well as vaccination and translational research [26,27,28]. One of the key elements of this evaluation approach is the use of narrow band filters for excitation and emission. The commonly used fluorophores FITC, Cy3 and Cy5 exhibit significant spectral overlap, but by use of specific filters bleed-over between signals can be reduced to inconsequential levels. Such filters are widely available nowadays, and various FluoroSpot imagers can be readily expanded for the simultaneous use of multiple filters. Hence, the hardware required for complex FluoroSpot analysis does not limit the FluoroSpot technique to the detection of only two analytes.

In addition, the large antibody binding capacity of FluoroSpot plates (roughly 100 µg/well) generally allows the expansion of the number of tested cytokines per well, considering the average use of only 1–2 µg per well of coating antibody for a specific analyte. A review of the chemical features of the PVDF membranes applied for ELISpot and FluoroSpot assays has been given elsewhere [29], and demonstrates the effective binding mechanism of proteins by the PVDF membrane if pretreated with Ethanol, and their stabilization for prolonged times when the coating procedure is followed by the addition of proteins (as done by “blocking” as included in most Elispot protocols, or the addition of stabilizer solutions, as done for commercially available pre-coated plates). A hypothetical situation where a region on the membrane efficiently binds one capture mAb but not the two other capture mAbs, resulting in single stained spots rather than dual or triple stained spots, is highly unlikely. If the membrane displayed such drastic variation in protein binding across a well, results within e.g., triplicates would display unusual high variation. Elsewhere in this issue, Dillenbeck *et al.* describe a comparison between results obtained from single and triple FluoroSpot for IFN-ɣ, IL-17A and IL-22. The two methods yielded a high correlation with similar spot numbers as well as spot intensities and quality. This too supports the excellent binding capacity of PVDF membranes for multiple capture mAbs.

Currently there are commercial kits available that test the secretion pattern for three cytokines in one well simultaneously. Such triple cytokine FluoroSpot approach is reviewed elsewhere in this issue (Dillenbeck *et al.*). When looking at multiple secretion patterns in FluoroSpot with more than two cytokines, it becomes evident that plate evaluation with a two level approach is essential for accurate analysis. The analysis of secretion patterns for three cytokines in one well will reveal seven sub-populations of cells (three single secretors, three combinations of dual secretors, and one triple secretor) (Figure 7), which will be impossible to separate by spot color. Furthermore, for every additional fluorophore introduced the number of potential subpopulations will roughly double, which in the case of a fourth analyte will require the software to identify 15 individual subpopulations (four single secretors, six combinations of dual secretors, four combinations of triple secretors, and one quadruple secretor). Hence the future of FluoroSpot has the potential to offer an extensive amount of data related to cytokine secretion patterns, while being performed in a similar straight-forward format as the conventional single color ELISpot.

The main current limitation for more complex FluoroSpot assays is the availability of high quality detection reagents, with high sensitivity and photostability. The same principles used for the analysis of two and three color FluoroSpot assays can be adapted to handle additional analytes in assays with more colors. Considering that a FluoroSpot assay evaluating two or three analytes simultaneously is principally as easy and straightforward to perform as a single color enzymatic ELISpot assay and exhibits comparable sensitivity, it can only be a question of time until complex FluoroSpot analysis will enter the market on a wide scale [30]. The principles for data acquisition and analysis have already been developed, as described here, and are in place for further expansion of the FluoroSpot technology.
